# Identification of radiation responsive genes and transcriptome profiling via complete RNA sequencing in a stable radioresistant U87 glioblastoma model

**DOI:** 10.18632/oncotarget.25247

**Published:** 2018-05-04

**Authors:** Ninh B. Doan, Ha S. Nguyen, Hisham S. Alhajala, Basem Jaber, Mona M. Al-Gizawiy, Eun-Young Erin Ahn, Wade M. Mueller, Christopher R. Chitambar, Shama P. Mirza, Kathleen M. Schmainda

**Affiliations:** ^1^ Department of Neurosurgery, Medical College of Wisconsin, Milwaukee, WI, USA; ^2^ Department of Radiology, Medical College of Wisconsin, Milwaukee, WI, USA; ^3^ Department of Medicine, Hematology/Oncology, Medical College of Wisconsin, Milwaukee, WI, USA; ^4^ Biophysics, Medical College of Wisconsin, Milwaukee, WI, USA; ^5^ Faculty of Medicine, University of Damascus, Damascus, Syria; ^6^ Mitchell Cancer Institute, University of South Alabama, Mobile, AL, USA; ^7^ Department of Chemistry and Biochemistry, University of Wisconsin, Milwaukee, WI, USA

**Keywords:** glioblastoma, acid ceramidase, acid ceramidase inhibitors, carmofur, radioresistance

## Abstract

The absence of major progress in the treatment of glioblastoma (GBM) is partly attributable to our poor understanding of both GBM tumor biology and the acquirement of treatment resistance in recurrent GBMs. Recurrent GBMs are characterized by their resistance to radiation. In this study, we used an established stable U87 radioresistant GBM model and total RNA sequencing to shed light on global mRNA expression changes following irradiation. We identified many genes, the expressions of which were altered in our radioresistant GBM model, that have never before been reported to be associated with the development of radioresistant GBM and should be concertedly further investigated to understand their roles in radioresistance. These genes were enriched in various biological processes such as inflammatory response, cell migration, positive regulation of epithelial to mesenchymal transition, angiogenesis, apoptosis, positive regulation of T-cell migration, positive regulation of macrophage chemotaxis, T-cell antigen processing and presentation, and microglial cell activation involved in immune response genes. These findings furnish crucial information for elucidating the molecular mechanisms associated with radioresistance in GBM. Therapeutically, with the global alterations of multiple biological pathways observed in irradiated GBM cells, an effective GBM therapy may require a cocktail carrying multiple agents targeting multiple implicated pathways in order to have a chance at making a substantial impact on improving the overall GBM survival.

## INTRODUCTION

Glioblastoma (GBM), with an overall survival of less than 1.5 years and a 5-year survival rate of 5%, is the most common, malignant primary cancer of the central nervous system in adults, with an estimated 12,120 new diagnoses in the United States alone in 2016 [1–3]. The current standard treatment regimen for GBM consists of maximal safe surgical resection, followed by the Stupp regimen, which consists of radiation therapy combined with concomitant and adjuvant temozolomide [4, 5]. Despite this, recurrence—characterized by radioresistance—is inevitable [6, 7]. To elucidate the underlying mechanism of radioresistance, we recently established a stable, radioresistant GBM model and also presented evidence partly attributing the radioresistance of GBM cells to radiation-induced upregulation of tumor promoters acid ceramidase (*ASAH1*) and sphingosine-1-phosphate (S1P) [8, 9]. *ASAH1* is a lysosomal cysteine amidase that converts ceramides, which trigger senescence and apoptosis, into sphingosine and free fatty acids [10–17]. Subsequently, sphingosine is phosphorylated by sphingosine kinase 1 (*SPHK1*) or 2 (*SPHK2*) to form a tumor promoter, S1P [10–15]. However, the radiation effects on global gene expression at the messenger ribonucleic acid (mRNA) level in a stable radioresistant GBM model have never been investigated.

RNA sequencing (RNA-seq) has become a powerful technique for transcriptome profiling of cell lines of interest to explain phenotypic variations [18, 19]. This study used RNA-seq to reveal many crucial radiation-responsive genes that may enable GBM cells to acquire resistance to radiation, providing the essential basis for further investigations of the role of these differential mRNA expressions in acquiring radioresistance.

## RESULTS

### Irradiation of GBM cells induced differential expression of 1094 radiation-responsive genes

**Figure 1:**
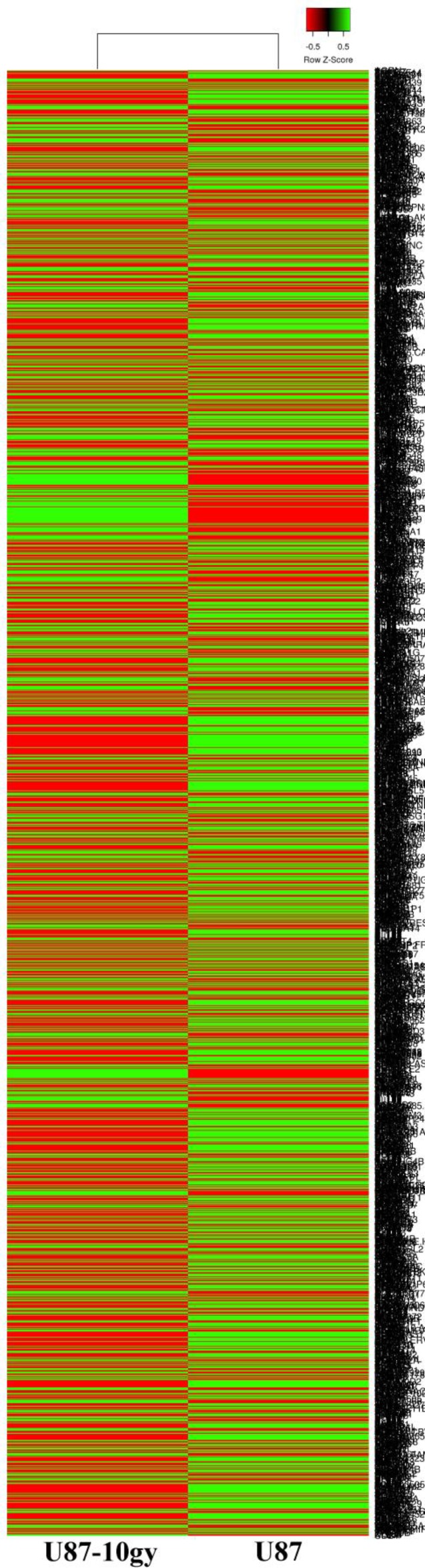
Clustered heat map of RNA-seq transcriptomes of U87 and U87-10gy cells U87 and U87-10gy cells were subjected to RNA sequencing. Experiments were performed in triplicates, and only genes with statically significant changes (~2000 genes) were utilized for the heat map. Green: relatively high expression, Red: relatively low expression. (*P* < 0.05)

We previously generated and described a stable radioresitant GBM model. Briefly, U87 cells received a total radiation of 10 Gy to generate radioresistant U87-10gy cells. Over the course of weeks, most cells died and less than ~1% of cells survived the irradiation. These radioresitant U87-10 gy cells were allowed to grow to confluence and were perpetuated for experiments. Total RNAs from U87 and U87-10 gy cells were harvested and subjected to further analysis. To screen for global mRNA changes following irradiation, we profiled transcriptomes of the control U87 cell line and its derivatives irradiated U87-10gy cells via RNA sequencing (Figure 1 and Supplementary Table 1). Using genes with twofold changes or greater with statistically significant values (*P* < 0.05) as criteria, we identified 1094 radiation responsive genes that were upregulated or downregulated in U87-10gy vs U87 cells. Among these 1094 radiation responsive genes, 427 were upregulated and 667 were downregulated (Supplementary Tables 2 and 3).

### Upregulation of genes promoting tumor aggressiveness and invasion following irradiation

In an effort to categorize genes based on their functions, we performed a gene ontology analysis. This analysis revealed that upregulated genes were enriched in inflammatory response, cell migration, positive regulation of epithelial to mesenchymal transition, angiogenesis, cell proliferation, cell growth, positive regulation of the canonical Wnt signaling pathway, response to hypoxia, activation of mitogen-activated protein kinase activity, metalloendopeptidase activity, cellular response to fibroblast growth factor stimulus, cellular response to transforming growth factor beta stimulus, cellular response to tumor necrosis factor, and ribonuclease A activity genes (Tables 1 and 2, and Supplementary Tables 2 and 4).

**Table 1:** Enriched gene ontology categories of differentially expressed genes following irradiation based on sets of statistically significant upregulated and downregulated genes (*P* < 0.05)

Differentially Expressed	Category	*P*-value
	GO:0007165: Signal transduction	6.90E–04
	GO:0006351: Transcription, DNA-templated	7.40E–03
	GO:0008152: Metabolic process	1.50E–04
	GO:0006915: Apoptotic process	6.60E–04
**Downregulated**	GO:0007155: Cell adhesion	1.50E–03
	GO:0072332: Intrinsic apoptotic signaling pathway by p53 class mediator	1.10E–02
	GO:0045746: Negative regulation of Notch signaling pathway	7.00E–02
	GO:2000406: Positive regulation of T-cell migration	4.10E–02
	GO:0010759: Positive regulation of macrophage chemotaxis	5.00E–02
	GO:0002457: T-cell antigen processing and presentation	9.60E–02
	GO:0002282: Microglial cell activation involved in immune response	9.60E–02
	GO:0006954: Inflammatory response	1.60E–04
	GO:0016477: Cell migration	2.90E–04
	GO:0010718: Positive regulation of epithelial to mesenchymal transition	4.90E–03
	GO:0001525: Angiogenesis	2.00E–02
	GO:0008283: Cell proliferation	2.30E–02
	GO:0016049: Cell growth	3.00E–02
**Upregulated**	GO:0090263: Positive regulation of canonical Wnt signaling pathway	4.10E–02
	GO:0001666: Response to hypoxia	2.90E–02
	GO:0000187: Activation of MAPK activity	7.50E–02
	GO:0004222: Metalloendopeptidase activity	5.70E–04
	GO:0004522: Ribonuclease A activity	8.00E–02
	GO:0071356: Cellular response to tumor necrosis factor	2.30E–04
	GO:0044344: Cellular response to fibroblast growth factor stimulus	2.50E–02
	GO:0071560: Cellular response to transforming growth factor beta stimulus	1.90E–02

Experiments were performed in triplicates.

**Table 2:** Upregulated genes of selected enriched gene ontology categories following irradiation are shown based on sets of statistically significant changes (*P* < 0.05)

Gene Ontology Category	*P*-value	Fold change	Gene symbol	Gene description
GO:0010718:Positive regulation of epithelial to mesenchymal transition	4.90E–03	2.32	*BAMBI*	BMP and activin membrane bound inhibitor
		2	*GLIPR2*	GLI pathogenesis related 2
		2.23	*AXIN2*	axin 2
		4.78	*COL1A1*	collagen type I alpha 1 chain
		2.79	*TGFB3*	transforming growth factor beta 3
GO:0001525: Angiogenesis	2.00E–02	2.46	*CXCL8*	C-X-C motif chemokine ligand 8
		5.1	*EPHB3*	EPH receptor B3
		2.2	*EPHB4*	EPH receptor B4
		5.79	*ACKR3*	atypical chemokine receptor 3
		2	*CSPG4*	chondroitin sulfate proteoglycan 4
		2.48	*COL8A1*	collagen type VIII alpha 1 chain
		3	*NRXN3*	neurexin 3
		3.16	*NDNF*	neuron derived neurotrophic factor
		3.68	*NRP2*	neuropilin 2
		2.611	*SERPINE1*	serpin family E member 1
		3.2	*ZC3H12A*	zinc finger CCCH-type containing 12A
GO:0016049: Cell growth	3.00E–02	14.44	*ROS1*	ROS proto-oncogene 1, receptor tyrosine kinase
		3.07	*EDN1*	endothelin 1
		2.43	*IL7R*	interleukin 7 receptor
		3.16	*NDNF*	neuron derived neurotrophic factor
		2.79	*TGFB3*	transforming growth factor beta 3
GO:0008283: Cell proliferation	2.30E–02	2.18	*E2F8*	E2F transcription factor 8
		4.84	*ERG*	ERG, ETS transcription factor
		14.44	*ROS1*	ROS proto-oncogene 1, receptor tyrosine kinase
		4.47	*ALK*	anaplastic lymphoma receptor tyrosine kinase
		2.23	*AXIN2*	axin 2
		3	*CDC25A*	cell division cycle 25A
		2.02	*CSPG4*	chondroitin sulfate proteoglycan 4
		4.19	*CYP1A1*	cytochrome P450 family 1 subfamily A member 1
		3.23	*DLX5*	distal-less homeobox 5
		2.14	*FSCN1*	fascin actin-bundling protein 1
		2.76	*FGF5*	fibroblast growth factor 5
		2.62	*GRPR*	gastrin releasing peptide receptor
		5.56	*MYH10*	myosin heavy chain 10
		2.47	*PDK1*	pyruvate dehydrogenase kinase 1
		2.25	*UHRF1*	ubiquitin like with PHD and ring finger domains 1
GO:0004222: Metalloendopeptidase activity	5.70E–04	3	*ADAM12*	ADAM metallopeptidase domain 12
		2.79	*ADAM19*	ADAM metallopeptidase domain 19
		2.59	*ADAMTS1*	ADAM metallopeptidase with thrombospondin type 1 motif 1
		2.73	*ADAMTS14*	ADAM metallopeptidase with thrombospondin type 1 motif 14
		2.25	*BMP1*	bone morphogenetic protein 1
		3.15	*MMP12*	matrix metallopeptidase 12
		5.55	*MMP3*	matrix metallopeptidase 3
		5.69	*MMP7*	matrix metallopeptidase 7
		2.35	*MME*	membrane metalloendopeptidase
		2.4	*TSHZ2*	teashirt zinc finger homeobox 2
GO:0001666: Response to hypoxia	2.90E–02	5.69	*ABAT*	4-aminobutyrate aminotransferase
		2.73	*BNIP3*	BCL2 interacting protein 3
		5.6	*CA9*	carbonic anhydrase 9
		4.17	*CYP1A1*	cytochrome P450 family 1 subfamily A member 1
		3.84	*EGLN3*	egl-9 family hypoxia inducible factor 3
		2.25	*LOXL2*	lysyl oxidase like 2
		2.72	*MUC1*	mucin 1, cell surface associated
		10.81	*POSTN*	periostin
		2.79	*TGFB3*	transforming growth factor beta 3
		1.80	*HIF-1α*	Hypoxia-inducible factor 1-alpha
GO:0016477: Cell migration	2.90E–04	2.32	*BAMBI*	BMP and activin membrane bound inhibitor
		2.39	*EPHA3*	EPH receptor A3
		5.1	*EPHB3*	EPH receptor B3
		4.84	*ERG*	ERG, ETS transcription factor
		2.95	*WWC1*	WW and C2 domain containing 1
		2.06	*BDKRB1*	bradykinin receptor B1
		2	*CSPG4*	chondroitin sulfate proteoglycan 4
		2.41	*COL5A1*	collagen type V alpha 1 chain
		2.14	*FSCN1*	fascin actin-bundling protein 1
		3.66	*LCP1*	lymphocyte cytosolic protein 1
		3.3	*PODXL*	podocalyxin like
		4.48	*PSG2*	pregnancy specific beta-1-glycoprotein 2
		2.83	*SDC1*	syndecan 1

Experiments were performed in triplicates.

Many genes involved in enhancement of tumor aggressiveness and invasion were significantly induced by irradiation (Table 2 and Supplementary Table 2). For example, the upregulation of *BNIP3, MMP3, MMP7, MMP15, TGFBI, NOTCH2, AKT1, AKT3, TNFAIP3, RRM2, CXCL8, FOXM1, HMOX1, PRMT5, KDM2B, CERS6, SPHK1, ZBTB18,* and *PDK1* have been linked to radioresistance and increased aggressiveness of irradiated GBM cells [20–40]. In their study of irradiated GBM cells, Maachani *et al.* revealed *FOXM1* confers radioresistance in GBM cells [31]. *AKT1* and *PDK1* induce resistant to chemotherapy such as temozolomide [39, 40]. In addition, *AKT1* plays a major role in DNA repair of double-stranded breaks [40].

### Tumor suppressor, immune response, P-53-dependent apoptosis, and cell adhesion genes are enriched in the pool of downregulated genes

The gene ontology analysis was conducted to analyze differentially expressed genes. Compared with control U87 cells, downregulated genes in irradiated U87-10gy cells were enriched in signal transduction, transcription DNA-templated, metabolic pathway, apoptotic process, cell adhesion, intrinsic apoptotic signaling pathway by p53 class mediator, positive regulation of T-cell migration, positive regulation of macrophage chemotaxis, T-cell antigen processing and presentation, and microglial cell activation involved in immune response genes (Tables 1 and 3, and Supplementary Tables 3 and 5).

**Table 3:** Downregulated genes of selected enriched gene ontology categories following irradiation are shown based on sets of statistically significant changes (*P* < 0.05)

Gene Ontology Category	*P*-value	Fold change	Gene symbol	Gene description
GO:0006915: Apoptotic process	6.60E–04	4.30E–01	*BBC3*	BCL2 binding component 3
		7.90E–02	*DCC*	DCC netrin 1 receptor
		1.90E–01	*PYCARD*	PYD and CARD domain containing
		4.10E–01	*TRAF5*	TNF receptor associated factor 5
		1.30E–01	*XAF1*	XIAP associated factor 1
		4.10E–01	*BEX2*	brain expressed X-linked 2
		2.80E–01	*CASP1*	caspase 1
		1.60E–01	*CTSH*	cathepsin H
		2.30E–01	*C5AR1*	complement C5a receptor 1
		1.50E–01	*ELMO1*	engulfment and cell motility 1
		4.50E–01	*IL1B*	interleukin 1 beta
		2.60E–01	*MAP2K6*	mitogen-activated protein kinase kinase 6
		4.70E–01	*NR4A1*	nuclear receptor subfamily 4 group A member 1
		3.70E–01	*PMAIP1*	phorbol-12-myristate-13-acetate-induced protein 1
		1.10E–03	*SFRP2*	secreted frizzled related protein 2
		4.80E–01	*YARS*	tyrosyl-tRNA synthetase
GO:0072332: Intrinsic apoptotic signaling pathway by p53 class mediator	1.10E–02	3.50E–01	*PERP*	PERP, TP53 apoptosis effector
		1.90E–01	*PYCARD*	PYD and CARD domain containing
		3.70E–01	*PMAIP1*	phorbol-12-myristate-13-acetate-induced protein 1
		4.40E–01	*ZMAT1*	zinc finger matrin-type 1
		2.60E–01	*ZNF385D*	zinc finger protein 385D
GO:2000406: Positive regulation of T-cell migration	4.10E–02	1.90E–01	*PYCARD*	PYD and CARD domain containing
		1.40E–01	*TNFRSF14*	TNF receptor superfamily member 14
		2.50E–01	*ITGA4*	integrin subunit alpha 4
GO:0002457: T-cell antigen processing and presentation	9.60E–02	3.80E–01	*ICAM1*	intercellular adhesion molecule 1
		1.30E–01	*RFTN1*	raftlin, lipid raft linker 1
GO:0002282: Microglial cell activation involved in immune response	9.60E–02	4.70E–01	*IL33*	interleukin 33
		4.90E–01	*TLR3*	toll like receptor 3
GO:0010759: Positive regulation of macrophage chemotaxis	5.00E–02	8.50E–02	*CMKLR1*	chemerin chemokine-like receptor 1
		2.40E–01	*C5AR1*	complement C5a receptor 1
		8.50E–02	*TNFSF18*	tumor necrosis factor superfamily member 18

Experiments were performed in triplicates.

Downregulation of tumor suppressor genes after irradiation has been described as a major mechanism for GBM cells to enhance their survival [41]. Highlighting the negative association with survival, RNA-seq revealed downregulation of the following representative well-known genes involved in the negative regulation of cell survival: *S1PR1, PARP15, HOXA11*, and *ADGRG1* [42–45]. Improved prognosis in patients with GBMs has been associated with the high expression of *S1PR1*, suggesting its function as a tumor suppressor [42]. GBMs become less susceptible to radiation and chemotherapy when *HOXA11* expression is suppressed [44]. The loss of *ADGRG1*'s function promotes radioresistance of glioma-initiating cells [45].

## DISCUSSION

Investigators have shown that irradiation of GBM cells can transform them into a more malignant form [6, 7]. In their transcriptome analysis of glioma, Ma *et al.* describe that radioresistance of gliomas within hours following irradiation may be due to the inactivation of early proapoptotic molecules and late activation of antiapoptotic genes [41]. It is important to note that in their study, the transcriptome analysis was performed within hours (6–48 hours) following radiation, while in our study, the analysis was done in a stable radioresistant GBM model that we recently developed [8]. Since their study performed analysis within hours following radiation, their findings likely include cells that would not survive radiation long-term. On the other hand, our stable radioresistant GBM model selected out these cells, and our final RNA-seq involved only truly radioresistant cells. Consistent with the Ma *et al.* study, the aberrant gene expressions observed in irradiated U87-10gy cells were enriched in genes involved in enhancing tumor malignancy and invasion. Similar to the Ma *et al.* study, we also found upregulation of antiapoptotic genes *BNIP3* and *SOD2* in irradiated U87-10gy cells. Exclusively enriched in upregulated genes are positive regulation of epithelial to mesenchymal transition, metalloendopeptidase activity genes, and response to hypoxia genes. Upregulated metalloproteases mRNA expressions include *MME, MMP2, MMP3, MMP7, MMP12, ADAM9,* and *ADAM12*. Metalloproteases have been strongly implicated in encouraging tumor invasion and metastasis of many cancers by degrading the extracellular protein matrix [21, 22]. Epithelial to mesenchymal transition, a process that overlaps with the acquirement of stem cell properties characterized by increased cell motility and resistance to chemo- and radiotherapy, is an important inducer of cancer stem-like phenotypes and is associated with an aggressive phenotype in glioma [46, 47]. Epithelial to mesenchymal transition is typically induced by *TGFB3*, which was also upregulated in irradiated U87-10gy cells [47, 48]. Hypoxia, which is frequent in GBM, is a major inducer of epithelial to mesenchymal transition as well and is also a main promoter of GBM invasion [47, 49]. In GBM, hypoxia-inducible factor 1-alpha (HIF-1α) and carbonic anhydrase 9 expressions are induced by hypoxia and promote angiogenesis, migration, cell survival, proliferation, epithelial to mesenchymal transition, and radio- and chemoresistance [49, 50]. In line with this, we found HIF-1α and carbonic anhydrase 9 expressions upregulated in irradiated U87-10gy cells (Table 2).

Downregulated genes were enriched in tumor suppressor, positive regulation of immune response, P-53 dependent apoptosis, and cell adhesion genes (Tables 1 and 3). On the basis of gene ontology, we uncovered 16 genes associated with apoptosis, 10 genes associated with positive regulation of immune response, and 29 genes associated with cell adhesion that were downregulated (Tables 1 and 3). Ma *et al.* suggest that suppressing apoptotic potential through gene expression regulation of irradiated cells helps explain the radioresistant nature of irradiated GBM cells [41]. Consistent with our findings, multiple studies have identified downregulation of some of the apoptotic genes discovered in this study to play major roles in attenuating GBM apoptosis, especially for *BBC3, DCC, BEX2, CASP1, IL1B,* and *SFRP2* [51–56]. For example, in their study of GBM cell migration and proliferation, investigators identified *BBC3* as a potent inhibitor of cell migration and proliferation in GBM [51]. Downregulation of *DCC* expression was suggested as an important marker in tumor malignancy and recurrence in astrocytic tumors [52]. To further enhance their survival, GBM cells produce an immunosuppressive microenvironment to escape immune surveillance [57]. One mechanism to achieve this objective is through the secretion of transforming growth factor B (TGF-β) to block T-cell activation and proliferation [57]. In addition to TGF-β, we uncovered many other downregulated genes involved in the activation of the immune system, especially genes mediating T-cell antigen processing and presentation, that may help with immune evasion of radioresistant GBM cells (Tables 2 and 3).

A previous study of our recently described radioresistant GBM model revealed that radiation-induced accumulation of the *ASAH1* protein level, as measured by immunoblotting, may enable GBM cells to survive radiation [8, 9]. However, RNA-seq data demonstrated no changes in the mRNA expression of *ASAH1* between U87 and U87-10gy cells. This discrepancy can be explained by the reported absence of the high degree of, or even negative correlation (~40% on average), between mRNA and protein expressions [19, 58–62]. Similar mRNA levels can produce varied levels of protein of interest depending on the regulations between the transcripts and protein product such as the ability of the cells to stabilize the mRNA, the rates of translation, the rates of protein degradation, etc. [19, 59, 60]. As seen in this study, irradiation of U87 cells resulted in significant gene expression changes, which may alter post-transcriptional regulation and ultimately affect the resultant protein expression level.

Therapeutically, with the global alterations of multiple biological pathways observed in irradiated GBM cells, an effective GBM therapy may require a cocktail carrying multiple agents targeting multiple implicated pathways in order to have a chance at making a substantial impact on improving the overall GBM survival. These findings of aberrantly expressed mRNAs following irradiation provide a crucial comprehensive starting point to understand the complex mechanism of radioresistance in GBM, and should be combined with immunoblotting or other techniques of direct measurement of protein levels to supply a more accurate picture of how cells can be altered to be radioresistant. Many mRNAs, whose expressions were altered in our radioresistant GBM model, have never before been reported to be associated with the development of radioresistant GBM and should be further investigated to understand their roles in radioresistance.

The absence of a major progress in the treatment of GBM is partly attributable to our poor understanding of both GBM tumor biology and the acquirement of treatment resistance in recurrent GBMs. Recurrent GBMs are characterized by their resistance to radiation. In this study, we used an established stable radioresistant GBM model to shed light on global mRNA expression changes after irradiation. Many mRNAs, the expressions of which were altered in our radioresistant U87 GBM model, have never before been reported to be associated with the development of radioresistant GBMs and should be concertedly further investigated to understand their roles in radioresistance.

## MATERIALS AND METHODS

### Reagents and cells

The U87 and U87-10gy glioblastoma cell lines were cultured in Eagle's minimum essential medium containing 10% (v/v) fetal bovine serum. Culture medium materials were obtained from Life Technologies, Inc. (Grand Island, NY, USA).

### RNA library preparation and sequencing

RNA sequencing libraries were prepared using the TruSeq Stranded mRNA Library Prep Kit (Illumina, Inc., San Diego, CA, USA) according to the manufacturer`s protocol. The RNA concentration was measured with a NanoDrop 2000c spectrophotometer (Thermo Scientific Inc., Waltham, MA, USA). Integrity was assessed using an Agilent 2200 TapeStation instrument (Agilent Technologies, Santa Clara, CA, USA). Briefly, polyA mRNA from an input of 500 ng high-quality total RNA (RINe>8) was purified and fragmented. First strand complementary deoxyribonucleic acid (cDNA) syntheses were performed at 25° C for 10 minutes, 42° C for 15 minutes, and 70° C for 15 minutes, using random hexameres and ProtoScript II Reverse Transcriptase (New England BioLabs Inc., Ipswich, MA, USA). In a second strand cDNA synthesis, the RNA templates were removed and a second replacement strand was generated by incorporating deoxyuridine triphosphate (in place of deoxythymidine triphosphate, to keep strand information) to generate ds cDNA. The blunt-ended cDNA was cleaned up from the second strand reaction mix with beads. Next, the 3′ ends of the cDNA were adenylated and then indexing adaptors were ligated. Polymerase chain reactions (15 cycles of 98° C for 10 seconds, 60° C for 30 seconds, and 72° C for 30 seconds) were used to selectively enrich those DNA fragments that have adapter molecules on both ends and to amplify the amount of DNA in the library.

The libraries were quantified using the Promega QuantiFluor dsDNA System on a Quantus Fluorometer (Promega, Madison, WI). The size and purity of the libraries were analyzed using the High Sensitivity D1000 Screen Tape on an Agilent 2200 TapeStation instrument. The libraries were normalized, pooled, and subjected to cluster, and pair read sequencing was performed for 150 cycles on a HiSeq4000 instrument (Illumina, Inc., San Diego, CA, USA), according to the manufacturer's instructions.

### Generation of the stable radioresistant GBM model

The method and detail to generate the stable radioresistant GBM model was previously described by us [8]. U87 cell lines were grown to confluence and then irradiated with a Pantak HF320 X-ray machine (Agfa NDT Ltd., Reading, UK) operating at 300 kV at a dosage of 2.09 Gy/min to a total radiation dose of 10 Gy, to generate the U87-10gy cell lines. Following radiation, these irradiated cells were allowed to recover and to grow to confluence. The U87-10gy cell line was stable for continued passages for further studies.

### Gene ontology analysis

The gene ontology enrichment analysis was performed using DAVID Bioinformatics Resources 6.7, NIAIS/NIH (http://david.abcc.ncifcrf.gov/). The heatmap was performed with a web-based program (http://www.heatmapper.ca/expression).

## SUPPLEMENTARY MATERIALS TABLES













## Abbreviations

ASAH1: Acid cermadise
cDNA: Complementary deoxyribonucleic acid
DNA: Deoxyribonucleic acid
GBM: Giloblastoma
HIF-1α: Hypoxia-inducible factor 1-alpha
mRNA: Messenger ribonucleic acid
SIP: Sphingosine-1-phosphate
SPHK1: Sphingosine kinase 1
SPHK2: Sphingosine kinase 2
RNA: Ribonucleic acid
RNA-seq: RNA sequencing
TGF-β: Transforming growth factor B
