# Supplementary material for: Identification of radiation responsive genes and transcriptome profiling via complete RNA sequencing in a stable radioresistant U87 glioblastoma model
Source: Oncotarget. 2018 May 4;9(34):23532–42. doi: 10.18632/oncotarget.25247 (PMC5955095; doi:10.18632/oncotarget.25247)
Supplement: Supplementary file 1 [file oncotarget-09-23532-s001.pdf]

Identification of radiation responsive genes and transcriptome profiling via complete RNA sequencing in a stable radioresistant U87 glioblastoma model

SUPPLEMENTARY MATERIALS

Supplementary Table 1: Raw data of all differentially expressed genes are shown. $P < 0.05$ and experiments were performed in triplicates. See Supplementary_Table_1

Supplementary Table 2: Upregulated genes with 2-fold or higher changes are shown. $P < 0.05$ and experiments were performed in triplicates. See Supplementary_Table_2

Supplementary Table 3: Downregulated genes with 2-fold or higher changes are shown. $P < 0.05$ and experiments were performed in triplicates. See Supplementary_Table_3

Supplementary Table 4: Complete sets of upregulated genes of selected enriched gene ontology categories following irradiation are shown based on sets of statistically significant changes ($P < 0.05$). Experiments were performed in triplicates. See Supplementary_Table_4

Supplementary Table 5: Complete sets of downregulated genes of selected enriched gene ontology categories following irradiation are shown based on sets of statistically significant changes ($P < 0.05$). Experiments were performed in triplicates. See Supplementary_Table_5
